# GeneExpressScore Signature: a robust prognostic and predictive classifier in gastric cancer

**DOI:** 10.1002/1878-0261.12351

**Published:** 2018-09-28

**Authors:** Xiaoqiang Zhu, Xianglong Tian, Tiantian Sun, Chenyang Yu, Yingying Cao, Tingting Yan, Chaoqin Shen, Yanwei Lin, Jing‐Yuan Fang, Jie Hong, Haoyan Chen

**Affiliations:** ^1^ State Key Laboratory for Oncogenes and Related Genes Division of Gastroenterology and Hepatology Key Laboratory of Gastroenterology and Hepatology Ministry of Health Shanghai Institute of Digestive Disease Renji Hospital School of Medicine Shanghai JiaoTong University China

**Keywords:** gastric cancer, gene expression, LASSO, prognosis, signature

## Abstract

Although several prognostic signatures have been developed for gastric cancer (GC), the utility of these tools is limited in clinical practice due to lack of validation with large and multiple independent cohorts, or lack of a statistical test to determine the robustness of the predictive models. Here, a prognostic signature was constructed using a least absolute shrinkage and selection operator (LASSO) Cox regression model and a training dataset with 300 GC patients. The signature was verified in three independent datasets with a total of 658 tumors across multiplatforms. A nomogram based on the signature was built to predict disease‐free survival (DFS). Based on the LASSO model, we created a GeneExpressScore signature (GES_GC_) classifier comprised of eight mRNA. With this classifier patients could be divided into two subgroups with distinctive prognoses [hazard ratio (HR) = 4.00, 95% confidence interval (CI) = 2.41–6.66, P < 0.0001]. The prognostic value was consistently validated in three independent datasets. Interestingly, the high‐GES_GC_ group was associated with invasion, microsatellite stable/epithelial–mesenchymal transition (MSS/EMT), and genomically stable (GS) subtypes. The predictive accuracy of GES_GC_ also outperformed five previously published signatures. Finally, a well‐performed nomogram integrating the GES_GC_ and four clinicopathological factors was generated to predict 3‐ and 5‐year DFS. In summary, we describe an eight‐mRNA‐based signature, GES_GC_, as a predictive model for disease progression in GC. The robustness of this signature was validated across patient series, populations, and multiplatform datasets.

**Abbreviations**
AUC: area under the curve
CI: confidence interval
DFS: disease‐free survival
GC: gastric cancer
GES_GC_: GeneExpressScore signature
GEO: gene expression omnibus
GS: genomically stable
GSVA: gene set variation analysis
HR: hazard ratio
LASSO: least absolute shrinkage and selection operator
LNR: lymph node ratio
MSS/EMT: microsatellite stable/epithelial–mesenchymal transition
qRT‐PCR: quantitative reverse transcriptase–polymerase chain reaction
ROC: receiver operating characteristic
TCGA: The Cancer Genome Atlas

## 1 Introduction

Gastric cancer is the fourth most common malignancy worldwide despite the decreasing incidence over the past decades in western countries (Torre *et al*., 2015). In Asian countries, GC is still one of the leading reasons of cancer mortality. Most GC patients are identified at an advanced stage at the time of first diagnosis. Up to now, the established TNM staging system has been regarded as the best predictor of survival. Patients with stage I disease have a relatively good prognosis, whereas those with stage IV have a relatively poor prognosis. However, GC with the same stage might also have different prognoses because of the inherent clinical and molecular diversities of this cancer (Noh *et al*., 2014; Stahl *et al*., 2015). Thus, new valuable and sufficient strategies are needed to predict prognosis and further guide individual treatment in GC.

Recent studies have provided numerous prognostic gene expression signatures for GC (Chen *et al*., 2005; Cho *et al*., 2011; Kim and Rha, 2009; Leung *et al*., 2004; Setoguchi *et al*., 2011; Takeno *et al*., 2010; Wang *et al*., 2016b; Xu *et al*., 2010; Yamada *et al*., 2008; Yamaguchi *et al*., 2008). However, several crucial limitations should be noted. Some did not have enough sample sizes, and this might decrease the reliability of statistical conclusions (Xu *et al*., 2010; Yamada *et al*., 2008). Moreover, some signatures have not been applied to clinical practice mainly because of the lack of validation datasets to prove robustness (Kim and Rha, 2009; Yamaguchi *et al*., 2008). Furthermore, the statistical models applied in these studies might be unstable and fail to ensure low covariation among the numerous genes involved (Cho *et al*., 2011; Wang *et al*., 2016b). Last but not least, most signatures have not been successfully tested using more than two detection technologies (Chen *et al*., 2005; Kim and Rha, 2009; Leung *et al*., 2004; Takeno *et al*., 2010). Thus, we have focused on addressing these restrictions in this research. We have analyzed nearly 1000 GC specimens from different populations. Our conclusions were validated in three independent datasets, proving the robustness of the predictive value of the signature. Moreover, these datasets were processed by multiplatform technologies, including microarrays, RNA sequence, and qRT‐PCR. Finally, a least absolute shrinkage and selection operator (LASSO) Cox regression model was used to construct the signature. LASSO has been extensively applied to a Cox proportional hazard regression model for survival analysis with high‐dimensional data (Jiang *et al*., 2016; Zhang *et al*., 2013). It has been used for optimal selection of features in high‐dimensional microarray data with a robust prognostic value and low correlation among data to avoid overfitting (Tibshirani, 1997). Here, we report the development and validation of a GeneExpressScore signature, GES_GC_, for predicting survival of GC after surgery.

## 2 Materials and methods

### 2.1 Patients and tumor samples

In all, 978 GC samples from five independent datasets were analyzed in this research, including four datasets from Gene Expression Omnibus (GEO), one dataset from The Cancer Genome Atlas (TCGA), and one cohort from RenJi Hospital. To maintain consistency, all of the datasets from GEO were processed using the same chip platform (Affymetrix Human Genome U133 Plus 2.0 Array, Santa Clara, CA, USA) which has been extensively used for transcriptome analysis and has numerous advantages. This chip platform comprises 54 675 features and has high accuracy and reproducibility for each transcript. Initially, differential tests were performed on 10 paired GC and adjacent normal mucosa tissues (GSE79973). The training dataset consisted of gene expression data from 300 GC samples (GSE62254) (Cristescu *et al*., 2015). Similarly, validation dataset I was comprised an adequate number (192) of GC samples (GSE15459) (Ooi *et al*., 2009). Additionally, validation dataset II contained 406 GC samples accessed from TCGA (level III gene expression data, combining published and provisional GC samples, https://genome-cancer.ucsc.edu/). Finally, validation dataset III (Renji cohort) contained 60 fresh frozen primary GC samples consecutively collected at Shanghai Renji Hospital from January 2000 to January 2005.

The study was approved by the ethics committee of Shanghai Jiao Tong University School of Medicine, Renji Hospital. Written informed consent was obtained from patients enrolled in the study. The study conformed to the provisions of the Helsinki Declaration. None of the patients had received radiotherapy or chemotherapy prior to surgery. The tissue samples comprised at least 70% tumor cells. The median follow‐up time for survivors was 25.5 months (range 4–76).

### 2.2 Total RNA extraction and qRT‐PCR analysis

RNAiso Plus (Takara, Tokyo, Japan) was used to extract total RNA from 60 GC tissues (validation dataset III, Renji cohort) according to the manufacturer's protocol. Reverse transcription was performed using the PrimeScript RT Reagent Kit (Takara). An ABI Prism 7900HT Sequence Detection System (Applied Biosystems, Foster City, CA, USA) was applied to perform the quantitative PCR by using SYBR Premix Ex Taq II (Takara). The expression of eight genes of the GES_GC_ was normalized by ACTB (β‐actin), acting as an internal control. Expression levels of each gene were determined by the −▵*C*_T_ approach (▵*C*_T_ = *C*_T_ mRNA − *C*_T_ ACTB RNA). The primers of candidate genes are shown in Table S1.

### 2.3 Development and validation of the GES_GC_

Several signatures have been successfully constructed based on candidate biomarkers that are differentially expressed between tumor and adjacent normal tissues (Huang *et al*., 2012; Leung *et al*., 2004; Zhang *et al*., 2013). These mainly included a 35 miRNA‐based signature that could predict the prognosis of patients with stage II colon cancer and a seven‐gene signature that could predict the relapse and survival for early‐stage cervical carcinoma (Huang *et al*., 2012; Zhang *et al*., 2013). These studies indicated that some differentially expressed biomarkers in tumor and adjacent normal tissues might not only contribute to the development of cancer but also correlate with cancer prognosis. Therefore, to screen out the potential biomarkers, the gene expression profiling from GSE79973 was used for differential expression analysis based on the ‘Limma’ r package. Candidate genes were identified as significantly differentially expressed if the adjusted *P* value for multiple comparisons (false discovery rate, FDR) was less than 0.01.

LASSO is a comprehensive method for regression with high‐dimensional predictors (Tibshirani, 1997). LASSO has been extensively applied to the Cox proportional hazard regression model for survival analysis with high‐dimensional data (Zhang and Lu, 2007; Zhang *et al*., 2013). LASSO can also be used for optimal selection of variables in high‐dimensional microarray data with a robust prognostic value and low correlation among data to prevent overfitting. We used the LASSO Cox regression model to further screen out the most useful prognostic markers among the candidate genes in the training dataset. A multi‐mRNA‐based risk score, GES_GC_, was constructed and normalized to predict prognosis of GC. The ‘glmnet’ r package could be applied to perform the LASSO Cox regression model analysis. We selected the optimal cutoff value of normalized GES_GC_ using X‐tile plots based on the correlation with patient DFS. X‐tile plots provide a single and intuitive method to estimate the association between variables and survival. The X‐tile software can automatically select the optimal cutoff value based on the highest chi‐square value (minimum *P* value) identified by Kaplan–Meier survival analysis and log‐rank test (Camp *et al*., 2004). The x‐tile software version 3.6.1 was used to generate X‐tile plots (Yale University School of Medicine, New Haven, CT, USA).

The prognostic value of the GES_GC_ was further validated in another three independent datasets cross‐compared with three different platforms. For microarrays (training and validation dataset I), the background of the raw CEL files was adjusted using the robust multichip average (RMA) and then all the probesets were summarized and normalized to obtain single gene expression (Irizarry *et al*., 2017). The level III gene expression dataset of TCGA was directly accessed from UCSC Cancer Browser (validation dataset II). Specifically, as for the validation datasets III, individual expression levels of the genes consisting of the GES_GC_ were obtained by qRT‐PCR and the expression levels were assessed using ‐▵CT.

### 2.4 Statistical analysis

We assessed the correlation between GES_GC_ and clinicopathological features using independent‐samples *t*‐test and chi‐square test. Kaplan–Meier survival analysis and log‐rank test were used to estimate survival. A Cox proportional hazards model was used to perform standard univariate and multivariate analysis. Prediction error curves were used to compare the accuracy of survival models. The ‘pec’ r package can provide a set of functions for efficient computation of predicting error curves (Mogensen *et al*., 2012). We used ‘pec’ package to estimate the inverse probability of censoring weighting (IPCW) estimation of time‐dependent Brier score based on ten‐fold cross‐validation. The logistic and Cox regression coefficients were used to construct the nomogram. Calibration plots were generated to explore the performance characteristics of the nomogram. In the calibration plot, the *x*‐axis indicates predicted survival probability and the *y*‐axis indicates the actual freedom from DFS for the patients. The 45° line indicates an ideal performance of a nomogram that does a perfect outcome prediction corresponding with actual outcome. Time‐dependent receiver operating characteristic (ROC) analysis was performed to assess the predictive accuracy of the nomogram. Decision curve analysis was used to assess the clinical practicability of the nomogram. The ‘GSVA’ package was used to carry out differentially expressed gene sets analysis. All the statistical tests were performed with r software (version 2.15 and 3.22, Auckland, New Zealand) and sas software (version 8.02, Charlotte, NC, USA). Statistical significance was set at 0.05.

## 3 Results

### 3.1 Development and validation of the GES_GC_

**Figure 1:**
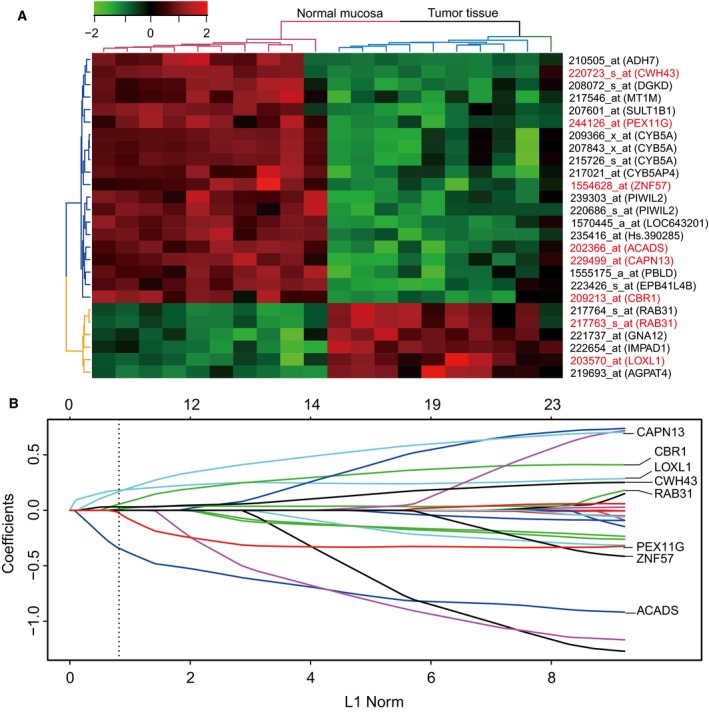
Construction of the GES_GC_, a prognostic classifier consisted of eight genes. (A) Heat map of mRNA expression profiles of the 26 differentially expressed in 10 paired gastric cancer and adjacent normal mucosa tissues. Rows represent genes, and columns represent patients. Pseudocolors represent transcript levels from low to high on a log 2 scale from −2 to 2, ranging from a low correlation power (dark, black) to high (bright, green, or red). (B) LASSO coefficient profiles of the 26 GC‐correlated genes. A dotted vertical line is drawn at the value identified by ten‐fold cross‐validation, where optimal λ results in eight nonzero coefficients.

**Figure 2:**
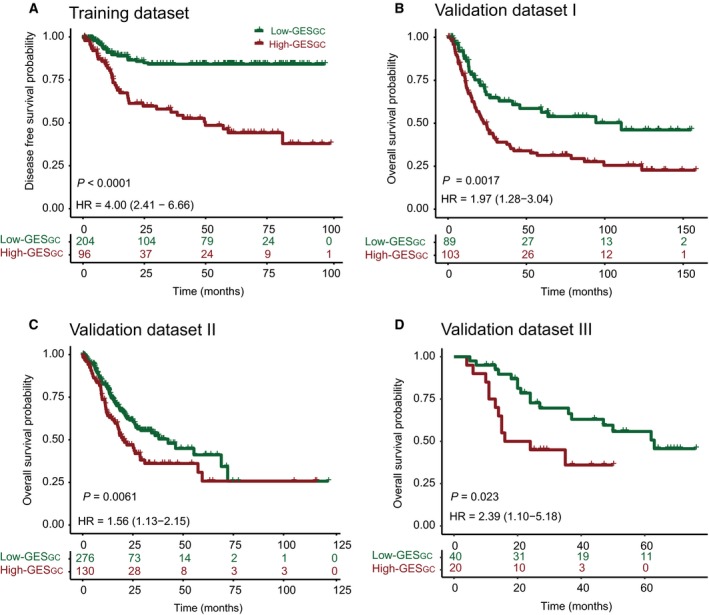
Kaplan–Meier estimates of survival based on the GES_GC_ in four datasets. (A) Training dataset. (B) Validation dataset I. (C) Validation dataset II. (D) Validation dataset III. The tick marks on the Kaplan–Meier curves represent the censored subjects. The differences between the two curves were determined by the two‐sided log‐rank test.

**Figure 3:**
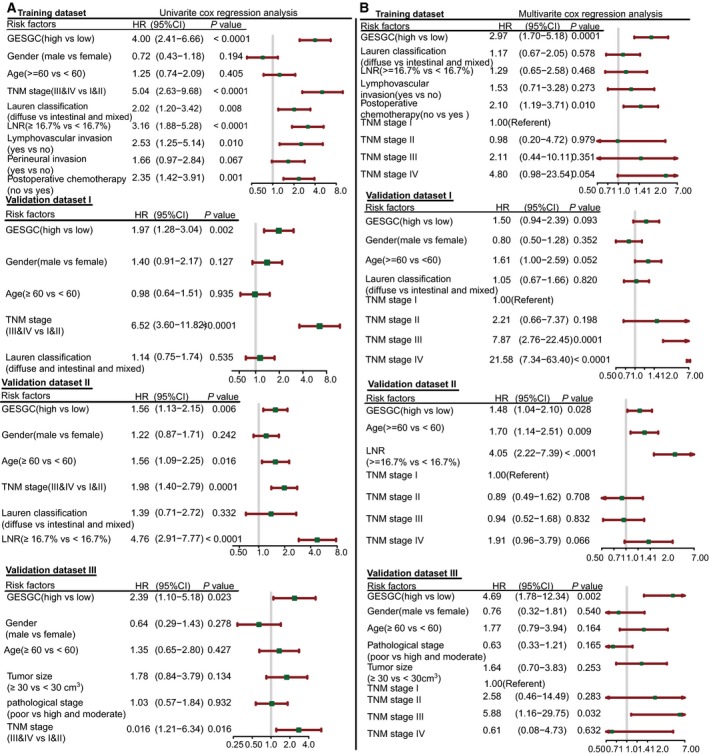
Univariate and multivariate based on GES_GC_ and clinical risk factors in four datasets. (A) Univariate Cox regression analysis. (B) Multivariate Cox regression analysis. Solid squares represent the hazard ratio (HR) of death, and close‐ended horizontal lines represent the 95% confidence intervals (CI). All *P* values were calculated using Cox regression hazards analysis. Abbreviations: LNR, lymph node ratio.

The study design is shown in Fig. S1A. Using the ‘Limma’ package, we identified 26 differentially expressed genes at probe levels in 10 paired tumor and adjacent normal mucosa tissues of GC (adjusted *P* value < 0.01) (Fig. 1A). Then, we used a LASSO Cox regression model to build a prognostic signature that selected eight out of the 26 genes identified in the training dataset (Figs 1B, S1B and Table S2). The gene expression of the eight genes had low correlations (Fig. S1C). Using the LASSO Cox regression model, we then derived a risk score for each patient based on the individual expression levels of the eight genes, namely GES_GC_ = (0.248345* expression level of CAPN13) + (0.124155* expression level of CBR1) + (0.19997* expression level of LOXL1) + (0.030862* expression level of CWH43) + (0.031894* expression level of RAB31) + (−0.15386* expression level of PEX11G) + (−0.03507* expression level of ZNF57) + (−0.45008* expression level of ACADS). Using X‐tile plots, patients in the training dataset were classified into high‐ or low‐GES_GC_ group with an optimum cutoff value of 0.4608 after GES_GC_ was normalized (Fig. S1D–F). The Kaplan–Meier survival analysis demonstrated that the two groups had significantly different outcomes (HR = 4.00, 95% CI = 2.41–6.66, *P *<* *0.0001; Fig. 2A). To confirm the robustness of the GES_GC_ classifier in different populations, it was further validated in three other independent datasets using the same cutoff point (validation I: HR = 1.97, 95% CI = 1.28–3.04, *P *=* *0.0017; validation II: HR = 1.56, 95% CI = 1.13–2.15, *P *=* *0.0061; validation III: HR = 2.39, 95% CI = 1.10–5.18, *P *=* *0.023; Fig. 2B–D). The stratification analyses indicated that the GES_GC_ classifier was a clinically and statistically prognostic model (Fig. S2A–J). Further, in the univariate Cox regression model, the GES_GC_ classifier was a strong variable correlated with prognosis in both training and validation datasets (Fig. 3A). After multivariate adjustment by clinical factors, the GES_GC_ classifier remained a powerful and independent prognostic factor in the training dataset, validation datasets II, III, and marginally in the validation dataset I (Fig. 3B).

### 3.2 The GES_GC_ and clinical‐molecule characteristics and pathway analysis

**Figure 4:**
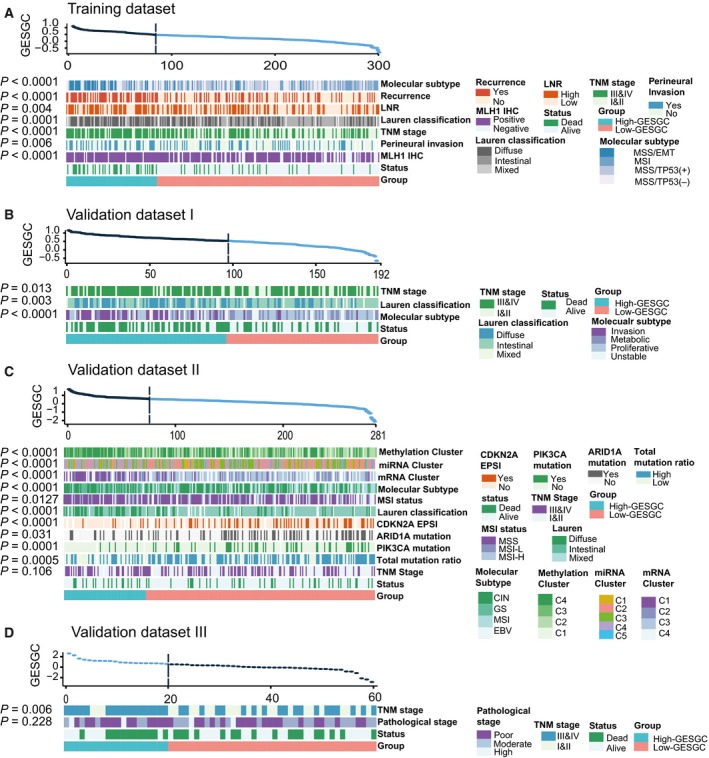
Heatmap of association between the GES_GC_ and clinical‐molecule characteristics in four datasets. (A) Training dataset. (B) Validation dataset I. (C) Validation dataset II. (D) Validation dataset III. The subjects were arranged based on the distribution of the GES_GC_ values from the highest to the lowest. All the *P* values were calculated using the chi‐square test. Abbreviations: LNR, lymph node ratio; IHC, immunohistochemistry; CIN, chromosomally unstable; GS, genomically stable; MSI, microsatellite instable; EBV, EBV‐infected; EMT, epithelial–mesenchymal transition; MSS, microsatellite stable; EPSI, epigenetic silencing.

Notably, we found that the distribution of several critical clinical‐molecule characteristics varied significantly between high‐ and low‐GES_GC_ groups (Fig. 4A–D, Table S3). We saw a substantially higher percentage of high TNM stage (III & IV) cases in the high‐GES_GC_ group than in the low‐GES_GC_ group of the training dataset (77.1% vs 48.5%, Table S3), and this condition was also overt in the validation datasets. Similar conclusions could be obtained for other clinical characteristics including lymph node ratio (LNR, 58.3% vs 40.7%), recurrence status (68.9% vs 32.8%), and perineural invasion status (37.5% vs 25.5%) in training dataset.

Interestingly, considerable overlaps were also observed between the GES_GC_ classifier and reported molecular subtypes. As regards the Lauren classification, 63.2% (60 in 95) high‐GES_GC_ group cases were classified as diffuse subtype in the training dataset as well as in validation datasets I (50%) and II (45.1%) (Fig. S3A). Further analysis demonstrated that the diffuse subtype had a shorter survival than other subtypes (*P *=* *0.023) and reached much higher median GES_GC_ value than the intestinal subtype (*P *<* *0.0001, Fig. S3B). The analyses for other two molecular subtypes also exhibited high similarities to the diffuse Lauren classification, including MSS/EMT subtype (Fig. S3C) and invasion subtype (Fig. S3D). Additionally, several integrative analysis clusters were consistently enriched in the high‐GES_GC_ group. In the high‐GES_GC_ group, 50.6% (39 in 77) of cases were mRNA cluster 1, 49.4% were miRNA cluster 4 (41 in 83), and 61.4% were DNA methylation cluster 4 (51 in 83) (Fig. S3A). Moreover, most of the genes highly expressed in mRNA cluster 1 were also highly expressed in the high‐GES_GC_ group (Fig. S3E). TCGA has reported that mRNA cluster 1 and miRNA cluster 4 had a substantial overlap and were strongly associated with GS subtype, and both of these clusters were enriched with a diffuse subtype (Cancer Genome Atlas Research, 2014). Our results showed that 33.7% of high‐GES_GC_ group cases were GS subtype (Fig. S3A). Although no significant prognostic differences were found among the four molecular subtypes (*P* = 0.894, ref. Sahm *et al*., 2017), the finding that the high‐GES_GC_ group was enriched with GS subtype might be of benefit for understanding the underlying molecular biological mechanisms of the GES_GC_ classifier.

We performed gene set variation analysis (GSVA) to explore differentially activated gene sets between high‐ and low‐GES_GC_ groups. The results implied that several metastasis‐, stemness‐, and adhesion‐associated gene sets were enriched in the high‐GES_GC_ group, and patients in the high‐GES_GC_ group were more likely to be resistant to cisplatin treatment (Fig. S3F). Furthermore, the correlation analysis suggested that there was a strong positive correlation between the GES_GC_ and these activated gene sets in the high‐GES_GC_ group (Fig. S3)G. To some extent, the above‐mentioned molecular characteristics may provide a reasonable interpretation of the prognostic value of the GES_GC_.

### 3.3 The GES_GC_ and published gene signatures

**Figure 5:**
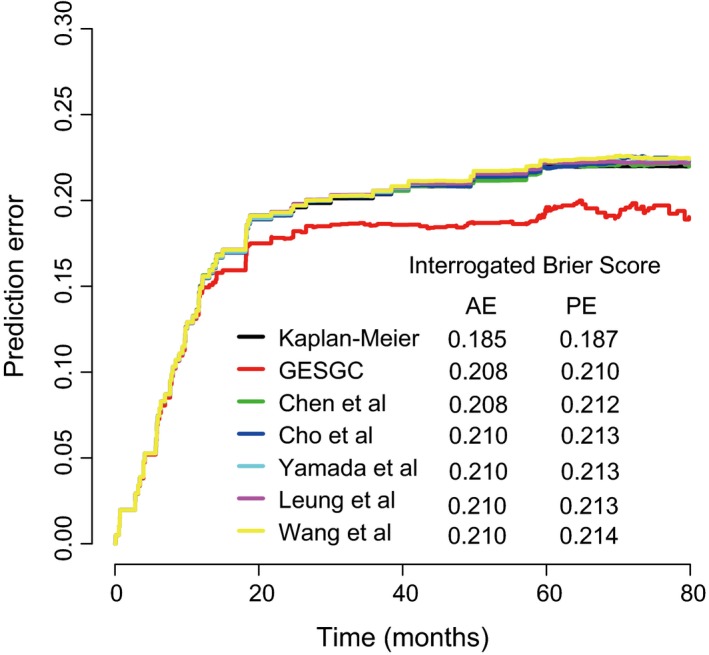
PEC analysis of GES_GC_ and published signatures in training dataset. Apparent error (AE) and ten‐fold cross‐validated cumulative prediction error (PE) were computed using Kaplan–Meier estimation as reference.

With the development of microarray technologies over the past decade, increasing prognostic gene expression signatures of GC have been published. A systematic study has summarized these published prognostic signatures in GC (Lin *et al*., 2015). Five reported signatures that fulfilled the following criteria were selected for comparison with the GES_GC_: (a) the total sample size was more than 50 and (b) the signature contained validation dataset(s). Complete details of the selected signature are provided in Table S4. We computed their accuracy using prediction error curves within training, validation I and II datasets. Prediction error over time was calculated using the Brier score. In general, the prediction error curve of the GES_GC_ was lower than the selected signatures that were reported. This implied that the GES_GC_ provided more precise prognostication of DFS outcomes (Fig. 5 and Fig. S4A,B).

### 3.4 Clinical utility of the GES_GC_

**Figure 6:**
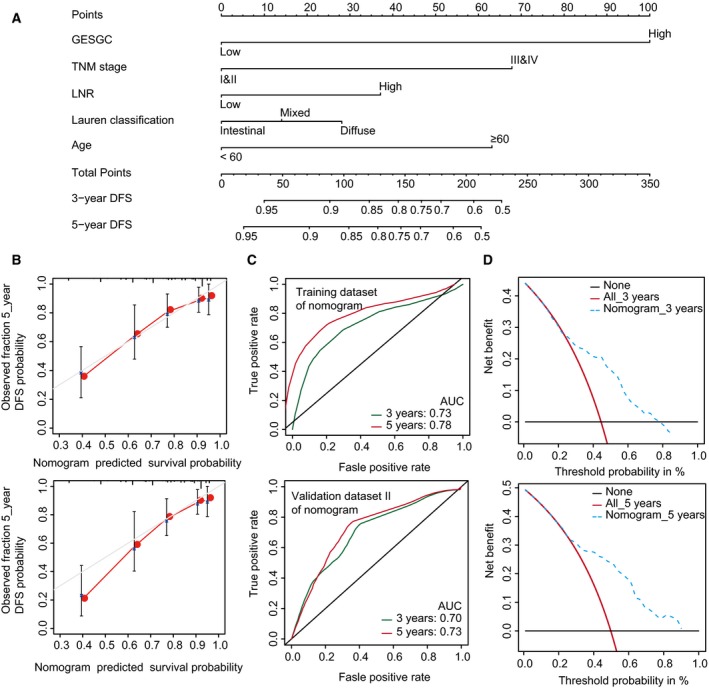
The developed nomogram to predict 3‐ and 5‐year DFS probability in GC. (A) The nomogram was constructed in the training dataset, with the GES_GC_ classifier, TNM stage, LNR, Lauren classification and age incorporated. (B) Calibration curve of the model in terms of agreement between predicted and observed 3‐ and 5‐year outcomes. Close‐ended vertical lines represent the 95% confidence intervals. The *x*‐axis indicates predicted survival probability, and the *y*‐axis indicates the actual freedom from DFS for the patients. The relative 45‐degree line indicates an ideal performance of a nomogram. (C) Time‐dependent ROC curves based on the nomogram for 3‐ and 5‐year DFS probability in the training dataset and validation dataset II. Not all of the clinical factors consist of nomogram could be obtained in validation I and III datasets. (D) Decision curve analysis of the nomogram. The *x*‐axis represents the percentage of threshold probability, and the *y*‐axis represents the net benefit. The black lines represent the assumption that no patients relapsed at 3 or 5 years. The red lines represent the assumption that all patients relapsed at 3 or 5 years. The blue dotted lines represent the prediction model of nomogram. The decision curve showed that if the threshold probability of a patient or doctor is more than 25%, using the nomogram to predict recurrent probability at 3 or 5 years adds more benefit than the treat‐all‐patients scheme or the treat‐none scheme. For example, if a recurrence probability at the point of 50% is used as a threshold, the net benefit of the nomogram is 0.16 and 0.24 for 3 and 5 years after surgery, respectively.

To provide a clinically correlated quantitative method that could predict the probability of 3‐ and 5‐year DFS in GC, a nomogram was generated by integrating the GES_GC_ and four clinicopathological risk factors (Fig. 6A). Calibration plots indicated that the nomogram performed well compared with an ideal model (Fig. 6B). The areas under the curve (AUC) at 3 and 5 years were 0.73 and 0.78 for the nomogram in the training dataset, respectively (Fig. 6C). The validation dataset II was used to test the predictive accuracy of the nomogram, and the AUCs at 3 and 5 years were 0.70 and 0.73, respectively (Fig. 6C). The decision curve showed that if the threshold probability of 3‐ and 5‐year DFS of a patient or doctor is more than 25%, using the nomogram to predict recurrent probability at 3 or 5 years adds more benefit than the treat‐all‐patients scheme or the treat‐none scheme (Fig. 6D).

## 4 Discussion

In the past decade, increasing technologies have been applied to human transcriptome analysis, including microarrays, high‐throughput RNA sequence, and qRT‐PCR. There are several well‐established platforms for microarrays, including Affymetrix, Illumina, and Agilent. To remain consistency, four datasets downloaded from the GEO were all used for the same chip platform (Affymetrix Human Genome U133 Plus 2.0 Array). Another two datasets were from RNA‐sequence and qRT‐PCR, respectively. There were nearly 1000 samples (*N *=* *978) included in this study. To our knowledge, this is the largest cohort used for constructing mRNA‐based prognostic signature in GC. Moreover, these specimens were from different populations including Europeans and Asians (Koreans, Singaporeans and Chinese). These cross‐platform and cross‐racial datasets were the basis of robustness of the signature presented here.

Prognostic models derived from high‐dimension data could carry a high risk of overfitting, which would decrease the significance of the predictor when applied to independent datasets. To overcome this limitation, we applied a Cox regression model with a LASSO penalty for shrinkage and selection of genes, facilitating selection of genes with a robust prognostic value, high expression variances, and low correlation among each other. Based on this method, we constructed an eight‐mRNA‐based prognostic classifier of GC that we have named GES_GC._

Recently, several novel multi‐mRNA‐based signatures in GC have been reported. Wang *et al*. generated a prognostic scoring system in GC based on a total of 53 genes (Wang *et al*., 2016b). The prognostic value was validated in an independent dataset. However, they did not try to reduce the number of genes and avoid redundancy in prognostic associations among these genes. Numerous genes may complicate the transfer to routine clinical trials. In our study, we used a LASSO Cox regression model to screen out a small set of genes to simplify such a transfer. According to the LASSO model, the eight selected genes obtained merely weak correlations in expression (median Pearson correlation 0.15). Another interesting ImmunoScore signature in GC was constructed by Jiang *et al*. (Jiang *et al*., 2016). They used a similar statistical model to select five out of 27 immune features.

However, several limitations should be noted. Firstly, the immune features involved did not represent all the GC‐associated immune features. Secondly, the expression levels of the immune biomarkers involved were based on immunohistochemistry conducted by pathologists. Thus, they probably could not be objectively evaluated in the clinical facility. Last but not least, all the specimens were from China, and little is known about the prognostic value in other races. We primarily performed differential expression analysis between cancerous and noncancerous GC samples to reduce thousands of genes to a representative set for further analysis. To assess the prognostic abilities of these signatures, we selected five signatures (sample size more than 50 and containing validation datasets) to compare with the GES_GC_ using predictive error curves. The predictive error curve has been widely used to evaluate and compare predictions in survival analysis (Gerds and Schumacher, 2007; Madhavan *et al*., 2012; Mogensen *et al*., 2012; Sahm *et al*., 2017). Ten‐fold cross‐validation was used to repeat data splitting, followed by estimation of the predictive error.

In addition to TNM staging system, several published classification systems have been generated for GC (Cancer Genome Atlas Research, N., 2014; Cristescu *et al*., 2015; Lauren, 1965). Intriguingly, our results indicated that the high‐GES_GC_ group was enriched with MSS/EMT, invasion, and GC subtypes. Previous studies have demonstrated that MSS/EMT subtype occurs at a significantly younger age and typically has a diffuse Lauren classification and lower number of mutation events compared with other MSS groups (Cristescu *et al*., 2015). The GS subtype includes diffuse classification and is associated with CDH1, RHOA mutations, CLDN18‐ARHGAP fusion, and cell adhesion (Cancer Genome Atlas Research, N., 2014). In addition, gene set variation analysis indicated that the high‐GES_GC_ group is more likely to be resistant to chemotherapy, especially cisplatin treatment. One interpretation might be that the high‐GES_GC_ group consists of a large proportion of EMT subtype. Previous studies have suggested that EMT could contribute to cancer drug resistance and metastasis after chemotherapy treatment, e.g. for pancreatic cancer (Arumugam *et al*., 2009), bladder cancer (McConkey *et al*., 2009), breast cancer (Huang *et al*., 2015), and gastric cancer (Wang *et al*., 2016a). This might in some way explain why the high‐GES_GC_ group has a worse survival compared with its counterpart.

Several genes involved in the GES_GC_ have been reported to be associated with human cancer, including LOXL1, RAB31 and CBR1. For example, LOXL1 contributes to the formation of crosslinks in collagens and elastin. It has been proved to be associated with several cancer types, including bladder cancer and juvenile papillary thyroid carcinoma, and may also be responsible for cisplatin resistance in non‐small‐cell lung cancer (Luzon‐Toro *et al*., 2015; Wu *et al*., 2007; Zhang *et al*., 2014). Several studies have demonstrated that RAB31 is correlated with prognosis in patients with breast, ovarian, liver cancer, and glioblastoma (Grismayer *et al*., 2012; Kotzsch *et al*., 2011; Serao *et al*., 2011; Sui *et al*., 2015). Specifically, RAB31 might promote hepatocellular carcinoma progression by inhibiting cell apoptosis induced by the PI3K/AKT/Bcl‐2/BAX pathway (Sui *et al*., 2015). CBR1 is correlated with doxorubicin resistance in human gastrointestinal cancer, and the efficacy of doxorubicin can be improved by inhibiting CBR1 in breast cancer treatment (Jo *et al*., 2017; Matsunaga *et al*., 2015). Although some of biological functions of the eight genes have not been reported in GC, they might be important targets for further biological and mechanistic investigation.

We have also noticed that there were no overlaps of these genes that consisted of pre‐existing five‐gene signature and GES_GC_. The possible reasons are as follows. Firstly, we should note that GC is a disease with high heterogeneity. Dysregulated genes involved with the biological process in individual GC patients might be different. Secondly, the datasets applied in these signatures were derived from different types of tissues such as GC of a specific stage, metastatic lymph nodes, and adjacent normal, or healthy tissue. The expression profiles of these tissues might also be distinctive. Thirdly, the datasets applied in these signatures were derived from different platforms, including cDNA microarray, transcripts microarray and exon array. The total numbers of genes detected by these platforms were different. This means that some of these platforms might not be able to detect some genes. Fourthly, although most of these signature genes were associated with survival (DFS or OS), different statistic models also determined that different genes might be included in these prognostic models.

Our study is limited because it is retrospective; validation of the GES_GC_ for each patient in a prospective, multicenter clinical trial is necessary. We also chose OS or DFS as the endpoint according to the clinical data accessed from the public databases. Although there are some differences between these two concepts, both of them are recognized as useful clinical endpoints. Additionally, to our knowledge, the clinical data used for construction of these signatures have not been available publically, preventing an assessment of the GES_GC_ in those GC samples. Finally, the mechanisms of the signature genes have not been clearly identified here, and experimental studies on these genes may provide important information to facilitate our understanding of their functional roles.

## 5 Conclusions

Implementation of molecular testing in clinical practice could refine prognosis prediction of GC. The GES_GC_ presented here is the first GC prognostic signature that is associated with molecular subtypes and successfully validated in national and international patient series, and among multiplatform generations. The GES_GC_ classifier is based on the expression levels of a small set of eight genes. It has shown its robustness of risk estimation of GC which hopefully can be applied to a prospective study for validation on individual GC patients.

## Authors' contributions

XZ, XT, TS, YL, J‐YF, JH, and HC participated in the design and performance of the study. XZ, XT, TS, YC, CY, YL, J‐YF, JH, and HC participated in analysis and interpretation of the data. XZ, XT, TY, CS, and HC performed statistical analysis. The manuscript was draft by XZ, XT, TS, and HC and reviewed by all authors. All authors read and approved the final manuscript.

## Data accessibility

The datasets supporting the conclusions of this article are available at the NCBI Gene Expression Omnibus repository (http://www.ncbi.nlm.nih.gov/projects/geo/): (a) GSE79973; (b) GSE62254; (c) GSE15459. Level III RNA sequence data of GC are available at UCSC Cancer Browser (https://genome-cancer.ucsc.edu/).

## Supporting information


**Fig. S1.** Construction and validation of GES_GC_.
**Fig. S2.** Subgroup analysis based on GES_GC_ classifier.
**Fig. S3.** Association between the GES_GC_ and clinical‐molecule characteristics and pathway analysis.
**Fig. S4.** PEC analysis of GES_GC_ and published signatures in validation datasets.
**Table S1.** Primers of eight genes and internal control for qRT‐PCR.
**Table S2.** Detailed description of the genes consisting of the GES_GC_.
**Table S3.** Clinical characteristics of patients in four datasets.
**Table S4.** Details of genes consisting of the five published signatures.
