# Supplementary material for: GeneExpressScore Signature: a robust prognostic and predictive classifier in gastric cancer
Source: Mol Oncol. 2018 Sep 28;12(11):1871–83. doi: 10.1002/1878-0261.12351 (PMC6210036; doi:10.1002/1878-0261.12351)
Supplement: Supplementary file 1 — Fig. S1. Construction and validation of GESGC. Fig. S2. Subgroup analysis based on GESGC classifier. Fig. S3. Association between the GESGC and clinical‐molecule characteristics and pathway analysis. Fig. S4. PEC analysis of GESGC and published signatures in validation datasets. Table S1. Primers of eight genes and internal control for qRT‐PCR. Table S2. Detailed description of the genes consisting of the GESGC. Table S3. Clinical characteristics of patients in four datasets. Table S4. Details of genes consisting of the five published signatures. [file MOL2-12-1871-s001.pdf]

GeneExpressScore Signature:

A robust prognostic and predictive classifier in gastric cancer

Xiaoqiang Zhu, Xianglong Tian, Tiantian Sun, Chenyang Yu, Yingying Cao, Tingting Yan, Chaoqin Shen, Yanwei Lin, Jing-Yuan Fang, Jie Hong & Haoyan Chen

State Key Laboratory for Oncogenes and Related Genes; Division of Gastroenterology and Hepatology; Key Laboratory of Gastroenterology and Hepatology, Ministry of Health; Renji Hospital, School of Medicine, Shanghai JiaoTong University; Shanghai Institute of Digestive Disease; 145 Middle Shandong Road, Shanghai 200001, China.

Corresponding author: Haoyan Chen, Jie Hong, Jing-Yuan Fang and Yanwei Lin. 145 Middle Shandong Road, Shanghai 200001, China. Fax: 021-63266027, Phone: 021-53882357. E-mails: yanwei_new@163.com (YL); jingyuanfang@sjtu.edu.cn (J-YF); jiehong97@sjtu.edu.cn (JH); Haoyan Chen, haoyanchen@sjtu.edu.cn (HC).

Running title: prognostic gene signature for gastric cancer

XZ, XT and TS contributed equally to this work.

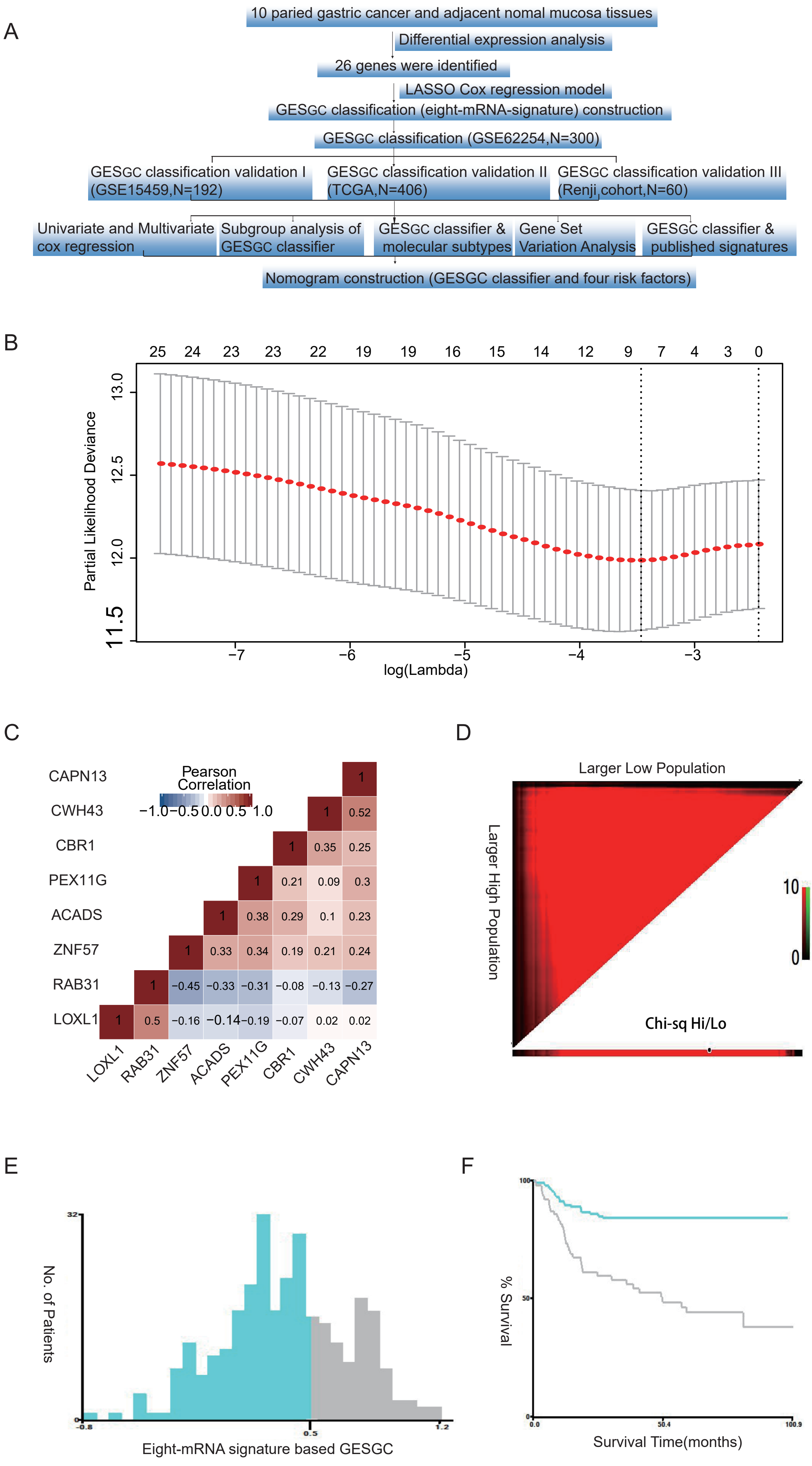

Supplementary Figure 1. Construction and validation of GESGC.
(A) Schematic overview of the work flow for the study. GC patients in the validation dataset III was from Renji Hospital. (B) Tuning parameter (λ) selection in the LASSO model used ten-fold cross-validation via minimum criteria. The solid vertical lines represent the partial likelihood deviance ± standard error (SE). The dotted vertical lines are drawn at the optimum values by minimum criteria and 1-SE criteria. A λ value of 0.03, with log (λ), -3.5 was chosen by ten-fold cross-validation via the 1-SE criteria. (C) The pearson correlation of expression signals between the eight genes in the GESGC classifier were generally weak (absolute value values, -0.45 to 0.52). (D-F) X-tile plots of the LASSO GESGC value in the training dataset. The optimum cutoff point highlighted by the black circle (D) is shown on a histogram of the entire dataset (E) and a Kaplan-Meier plot (F). Coloration of the plot indicates the strength of the correlation at each division, ranging from low (dark, black) to high (bright, green or red). Green indicates direct correlation between marker expression and survival, whereas red indicates inverse correlation (cutoff point = 0.4608, $\chi2 = 33.2558$, $P < 0.0001$).

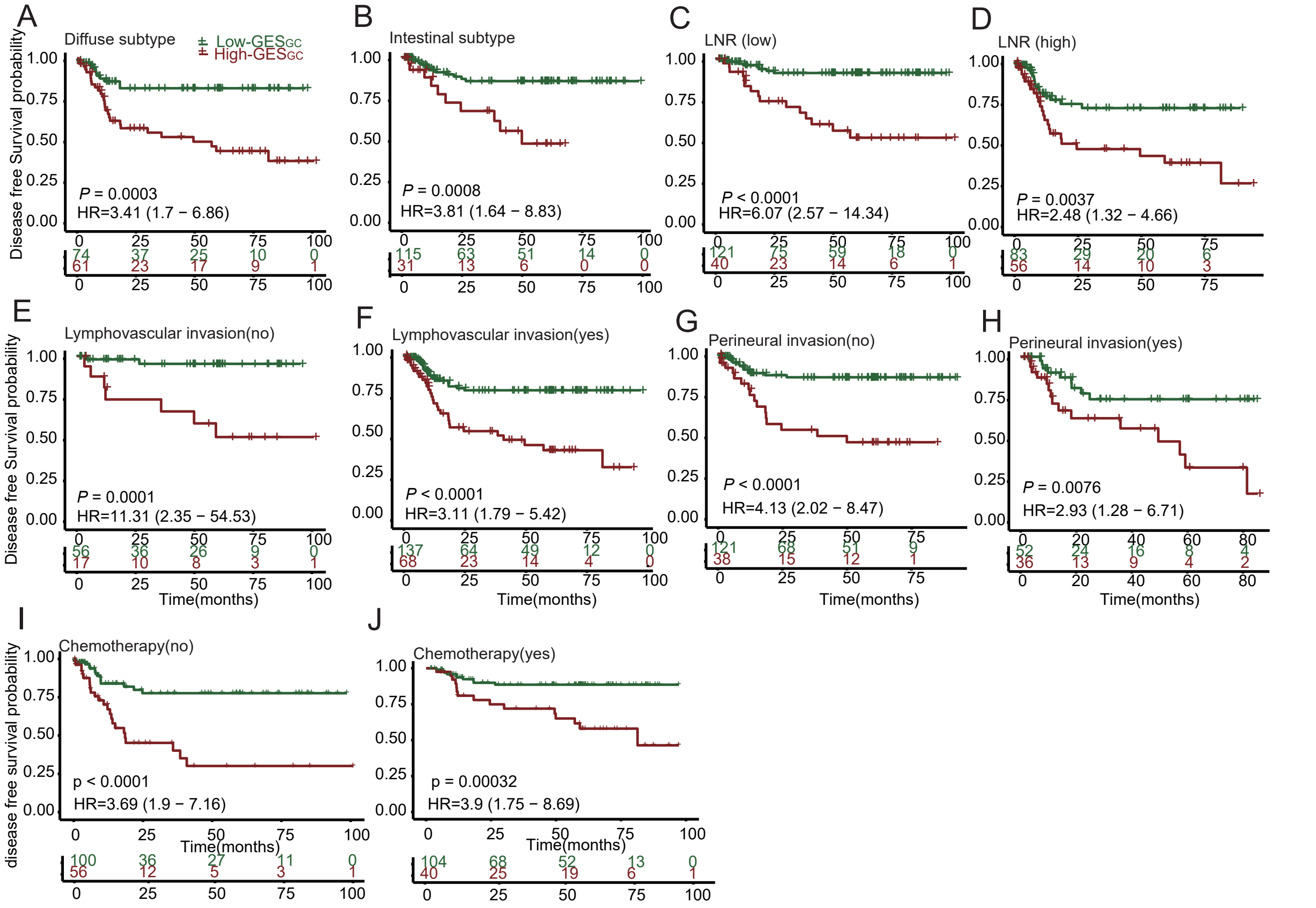

Supplementary Figure 2. Subgroup analysis based on GESGC classifier.

Kaplan-Meier survival analysis of DFS based on the GESGC stratified by clinicopathological risk factors in the training dataset, including Lauren classification, LNR, lymphovascular invasion status, perineural invasion status and adjuvant chemotherapy .The tick marks on the Kaplan-Meier curves represent the censored subjects. The differences between the two curves were determined by the two-side log-rank test.

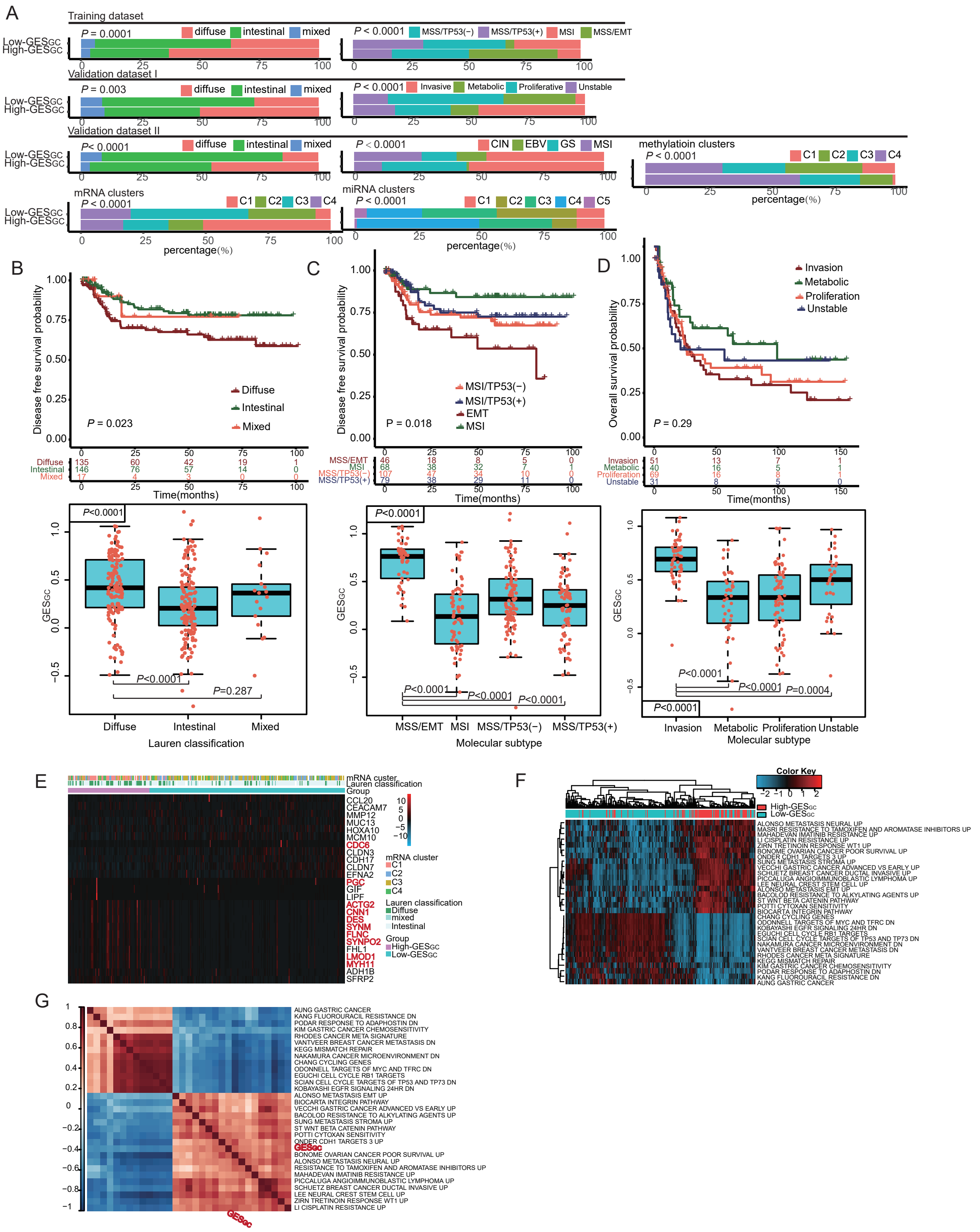

Supplementary Figure 3. Association between the GESGC and clinical-molecule characteristics and pathway analysis. (A) Percentage distributions of the clinical-molecule characteristics in training and validation datasets. All P values were calculated with chi-square test. (B-D) Kaplan-Meier survival analyses of DFS or OS based on the clinical-molecule subtypes and box plots of the GESGC distribution among the clinical-molecule subtypes in the training and validation datasets. The differences among curves were determined by log-rank test. Nonparametric Mann-Whitney Wilcoxon test was used to compare differences of GESGC among individual molecular subtypes.(E) Heat map of the mRNA expression profiles based on the GESGC stratification. Each subject was annotated by clinical characteristics including Lauren classification and mRNA expression cluster. Eight out of the genes that are highly expressed in cluster 1 are also highly expressed in high-GESGC group.(F) Gene set variation analysis (GSVA) of differentially activated gene sets based on the GESGC stratification. (G) Correlations matrix of expression signals between the GESGC and differentially activated gene sets. Heat maps were used for visualization of the corresponding results.

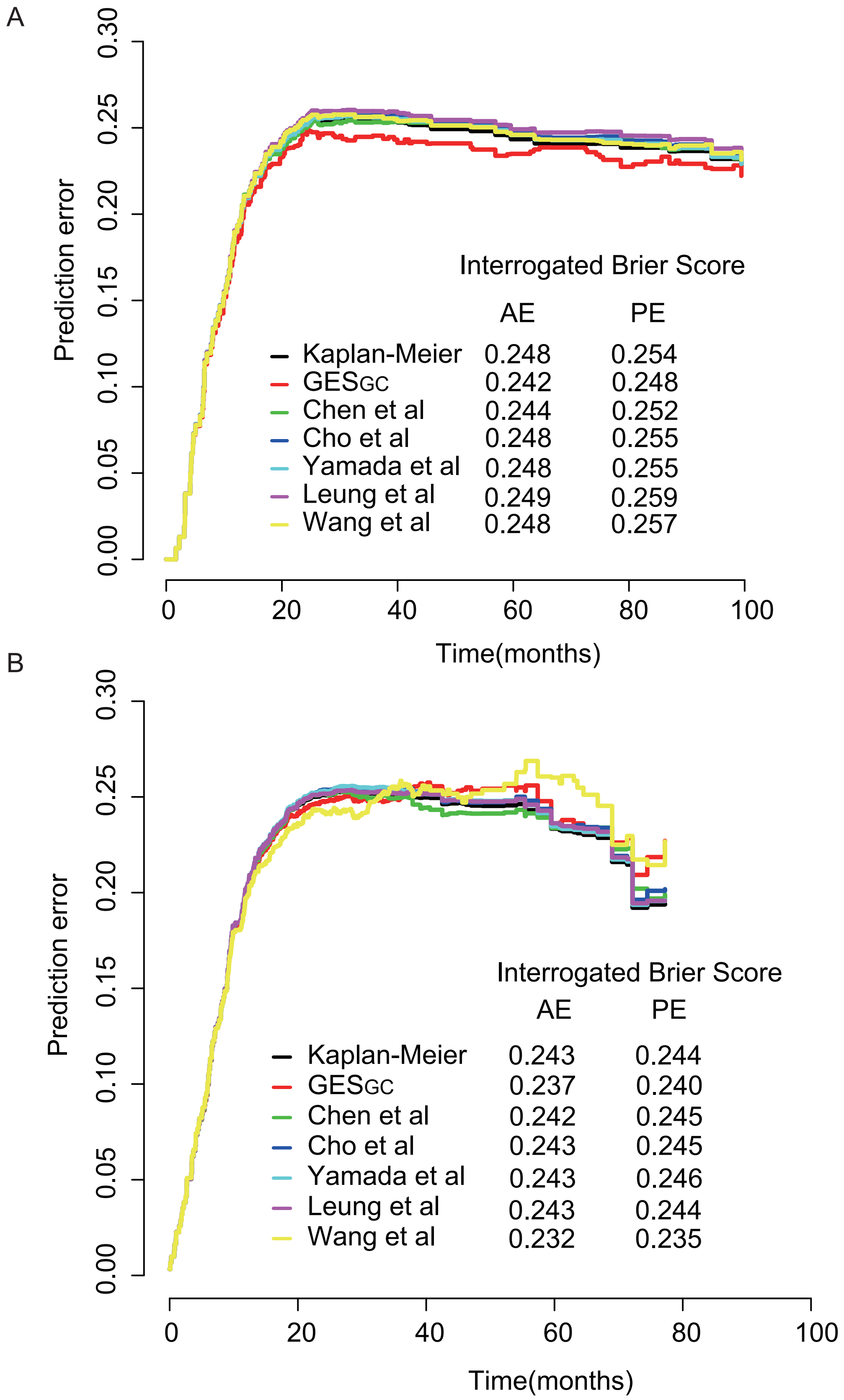

Supplementary Figure 4. PEC analysis of GESGC and published signatures in validation datasets. Apparent error (AE) and ten-fold cross-validated cumulative prediction error (PE) were computed using Kaplan-Meier estimation as reference.

Table S1. Primers of eight genes and internal control for qRT-PCR.

primers		5’ ⟶ 3’
ZNF57	Forward	TTGTGAAGGCTATGGCACTG
	Reverse	CTGGCATTTGATGGATGGCT
ACADS	Forward	GGACTTTGCCGAGAAGGAGT
	Reverse	TGAGCCGCTGGGAAGAGAT
	Forward	CCACCAGCATTACCACAGCA
LOXL1	Reverse	TTGAGGTTGCCGAAGTCACA
	Forward	AGATTGGCGTCACCGTTCTG
CBR1	Reverse	GCAGCAGGCATTCAGGAGGA
	Forward	ACGCTTCCACTTCACTCAAC
RAB31	Reverse	CAAATGCCGCTTGCTAACA
	Forward	GAGCATCACCTTCTTCCGTC
CWH43	Reverse	GGTTCCCAAAGTGTGTCACG
	Forward	CCTGGGAACTATGTTGTGGT
CAPN13	Reverse	GAAGATTCGGAGCAAGAACT
	Forward	TGGTTGGTGGAGTTCTGGTT
PEX11G	Reverse	AGTGGCTGAGTTGGGTGGAC
	Forward	ACTCTTCCAGCCTTCCTTCC
ACTB	Reverse	TCTCCTTCTGCATCCTGTCG

Table S2. Detailed description of the gene features consisting of the GES_{GC}.

Gene Symbol	Gene name	Associated diseases	LASSO coefficients
ZNF57	zinc finger protein 57	Gastric cancer	-0.03507400
ACADS	acyl-CoA dehydrogenase, C-2 to C-3 short chain	-	-0.45008396
LOXL1	lysyl oxidase-like 1	Bladder, juvenile papillary carcinoma and non-small lung cancer	0.19996980
CBR1	carbonyl reductase 1	Gastrointestinal cancer and breast cancer	0.12415508
RAB31	RAB31, member RAS oncogene family	Breast, ovarian, liver cancer and glioblastoma	0.03189390
CWH43	cell wall biogenesis 43 C-terminal homolog	-	0.03086192
CAPN13	calpain 13	-	0.24834474
PEX11G	peroxisomal biogenesis factor 11 gamma	-	-0.15386356

Table S3. Clinical characteristics of patients in four datasets.

	Training dataset			
	**Number of patients**	**Low-GES$_{GC}$**	**High-GES$_{GC}$**	***P* value**
**Age(years)**				
< 60	106	52(49%)	54(51%)	<0.0001
>=60	194	152(78%)	42(22%)	
**Sex**				
Male	199	143(72%)	56(28%)	0.044
Female	101	61(60%)	40(40%)	
**Tumor location**				
Antrum	150	107(71%)	43(29%)	0.65
Body	107	70(65%)	37(35%)	
Cardia, GE junction	30	19(63%)	11(37%)	
others	13	8(62%)	5(38%)	
**Lauren classification**				
Intestinal	146	115(79%)	31(21%)	0.0001
Diffuse	134	74(55%)	60(45%)	
Mixed	17	13(74%)	4(26%)	
**TNM stage**				
I & II	126	104(83%)	22(17%)	<0.0001
III & IV	172	98(57%)	74(43%)	
**EBV status**				
Positive	18	15(83%)	3(17%)	0.2
Negative	257	173(67%)	84(33%)	
**Lymphovascular invasion**				
Positive	205	137(67%)	68(33%)	0.051
Negative	73	56(77%)	17(23%)	
Missing	22	11(50%)	11(50%)	
**Lympy node ratio (%)**				
< 16.7	161	121(75%)	40(25%)	0.004
>= 16.7	139	83(60%)	56(40%)	

**Perineural invasion**				
Positive	86	52(60%)	36(40%)	0.006
Negative	159	121(76%)	38(24%)	
Missing	53	31(58%)	22(42%)	
**Recurrence status**				
Yes	125	63(50%)	62(50%)	<0.0001
No	157	129(82%)	28(18%)	
**Postoperative chemotherapy**				
Yes	144	104(72%)	40(28%)	0.132
No	156	100(64%)	56(36%)	
**Molecular subtype**				
MSS/TP53(-)	107	74(69%)	33(31%)	<0.0001
MSS/TP53(+)	79	63(80%)	16(20%)	
MSI	68	58(85%)	10(15%)	
MSS/EMT	46	9(20%)	37(80%)	
**MLH1 IHC**				
Positive	234	146(62%)	88(38%)	<0.0001
Negative	64	56(88%)	8(12%)	

		**Validation dataset I**		
	**Number of patients**	**Low-GES$_{GC}$**	**High-GES$_{GC}$**	***P* value**
**Age(years)**				
< 60	59	24(41%)	35(59%)	0.346
>=60	133	65(49%)	67(51%)	
**Sex**				
Male	124	57(46%)	67(54)%	0.88
Female	67	32(48%)	35(52%)	
**Lauren classification**				
Intestinal	98	57(58%)	41(42%)	0.003
Diffuse	75	24(32%)	51(68%)	
Mixed	18	8(44%)	10(56%)	
**TNM stage**				
I & II	60	36(60%)	24(40%)	0.013

III & IV	131	53(40%)	78(60%)	
**Molecualr subtype**				<0.0001
Invasive	51	4(8%)	47(92%)	
Metabolic	40	28(7%)	12(93%)	
Proliferative	69	44(64%)	25(36%)	
Unstable	31	13(42%)	18(58%)	

		**Validation dataset II**		
	**Number of patients**	**Low-GES$_{GC}$**	**High-GES$_{GC}$**	***P* value**
**Age(years)**				
< 60	122	78(64%)	44(36%)	0.199
>=60	287	202(70%)	85(30%)	
**Sex**				
Male	267	182(68%)	85(32%)	0.91
Female	147	101(69%)	46(31%)	
**Tumor location**				
Antrum	155	110(71%)	45(29%)	0.179
Body	143	103(72%)	40(28%)	
Cardia, GE junction	97	58(60%)	39(40%)	
others	19	12(63%)	7(37%)	
**Lauren classification**				
Intestinal	185	143(77%)	42(23%)	<0.0001
Diffuse	66	29(44%)	37(56%)	
Mixed	19	16(84%)	3(16%)	
**TNM stage**				
I & II	179	132(74%)	47(26%)	0.106
III & IV	210	139(66%)	71(34%)	
**EBV status**				
Positive	25	24(96%)	1(4%)	0.002
Negative	256	174(68%)	82(32%)	
**Recurrence status**				
Yes	31	18(58%)	13(42%)	0.088
No	159	117(74%)	42(26%)	
**Molecular Subtype**				

CIN	139	94(68%)	45(32%)	<0.0001
GS	56	27(48%)	28(52%)	
MSI	62	53(85%)	9(15%)	
EBV	25	24(96%)	1(4%)	
**Total Mutation Rate**				
High	138	85(62%)	53(38%)	0.0005
Low	138	112(81%)	26(19%)	
**Hypermutated**				
yes	62	52(84%)	10(16%)	0.016
No	214	145(68%)	69(32%)	
**TP53 mutation**				
Yes	130	95(73%)	35(27%)	0.555
No	146	102(70%)	44(30%)	
**PIK3CA mutation**				
Yes	56	51(91%)	5(9%)	0.0001
No	220	146(66%)	74(34%)	
**KRAS mutation**				
Yes	24	18(75%)	6(25%)	0.815
No	252	179(71%)	73(29%)	
**ARID1A mutation**				
YES	87	70(80%)	17(20%)	0.031
NO	189	127(67%)	62(33%)	
**RHOA mutation**				
YES	15	12(80%)	3(20%)	0.566
NO	261	185(71%)	76(29%)	
**Gene Expression Cluster**				<0.0001
C1	49	10(20%)	39(80%)	
C2	59	48(81%)	11(19%)	
C3	98	84(86%)	14(14%)	
C4	48	35(73%)	13(27%)	
**MicroRNA Expression Cluster**				

C1	31	22(71%)	9(29%)	<0.0001
C2	72	64(89%)	8(11%)	
C3	83	59(71%)	24(29%)	
C4	85	44(52%)	41(48%)	
C5	10	9(90%)	1(10%)	
**Copy Number Cluster**				
High	151	105(70%)	46(30%)	0.777
Low	128	91(71%)	37(29%)	
**Methylation Cluster**				
C1	27	26(96%)	1(4%)	<0.0001
C2	72	61(85%)	11(15%)	
C3	69	49(71%)	20(29%)	
C4	113	62(55%)	51(45%)	
**MSI status**				
MSS	178	117(66%)	61(34%)	0.0127
MSI_L	41	28(68%)	13(32%)	
MSI_H	62	53(85%)	9(15%)	
**CDKN2A_EPSI**				
yes	67	61(91%)	6(9%)	<0.0001
No	153	96(63%)	57(39%)	

	**Validation dataset III**			
	**Number of patients**	**Low-GES_{GC}**	**High-GES_{GC}**	***P* value**
**Age(years)**				
< 60	28	19(68%)	9(32%)	1.00
>=60	32	21(66%)	11(34%)	
**Sex**				
Male	34	30(88%)	11(12%)	0.146
Female	19	10(53%)	9(47%)	
**TNM stage**				
I & II	24	21(88%)	3(12%)	0.006
III & IV	36	19(53%)	17(47%)	
**Pathologic differentiation**				
High	4	2(50%)	2(50%)	0.228
Moderate	24	19(79%)	5(21%)	
Poor	32	19(59%)	13(41%)	

**Tumor size(cm^3)**				
< 30	27	18(67%)	9(33%)	1.00
>=30	33	22(67%)	11(33%)	

Table S4. Details of gene features consisting of the five reported signatures.

**Article**	**Genes**	**coefficients**
Leung et al(2004) Gastroenterology	CCL18	mean
Yamada et al(2008) Cancer Science	PDCD6	median
Cho et al(2011) Clin Cancer Res	CCL5	-0.0985
	CTNNB1	0.097
	EXOSC3	0.141
	LZTR1	-0.0618
	TOP2A	0.148
	TRANK1(LBA1)	-0.0898
Chen et al(2005) Journal of Clinical Oncology	CD36	0.833
	SLAM	-0.762
	PIM-1	-0.317
Wang et al(2016) Oncotarget	ABCE1	1.0363855
	ADH1C	0.764948
	ADNP	-1.053011
	ALDH6A1	-0.510668
	APOC1	0.981712
	APOE	-2.006557
	ATP13A3	0.883811
	BAZ1A	1.2705324
	BCAR3	2.3303943
	CAPRIN1	0.8505169
	CBFB	2.3508936
	CCT2	-0.579211
	CCT6A	-1.022031
	CEP55	-2.782475
	CHORDC1	-1.949418
	COL6A3	-0.697516
	CPXM1	-0.923039

CXCL1	0.6120864
CXCL10	3.8439059
ECHDC2	-0.908015
ENC1	0.4313284
EPHB4	0.7467178
ETFDH	2.344961
FGFR4	0.8526091
FHOD1	-2.518571
GABBR1	-2.005757
GART	-2.339531
INHBA	-0.517726
KAT2A	0.6196329
KLF4	-1.319586
LAMC2	-2.081441
LIMK1	1.6278243
LRRC41	2.5408068
MCM2	-3.728585
MMP14	0.9604701
NCL	-3.236286
NOL8	-1.315145
OSMR	0.5725561
P4HA1	-0.871475
PDP1	-1.73052
PGRMC2	-1.27645
PNO1	2.541787
PRC1	1.8534607
PRR7	2.7012202
PTGS1	0.7372285
SCNN1B	-0.501087
SLC12A9	-2.091898
SLC20A1	3.1705617
SMS	-1.355421
TCERG1	3.8908018
TGS1	1.3737803
TNFAIP2	-2.227492
TUBB	3.0609119
